# Characteristics and Outcomes of Dogs Admitted into Queensland RSPCA Shelters

**DOI:** 10.3390/ani7090067

**Published:** 2017-09-01

**Authors:** Megan Hemy, Jacquie Rand, John Morton, Mandy Paterson

**Affiliations:** 1 School of Veterinary Science, The University of Queensland, Gatton, QLD 4343, Australia; j.rand@uq.edu.au (J.R.); john.morton@optusnet.com.au (J.M.); mpaterson@rspcaqld.org.au (M.P.); 2 Australian Pet Welfare Foundation, Kenmore, QLD 4069, Australia; jacquie@petwelfare.org.au; 3 Jemora Pty Ltd., Geelong, VIC 3220, Australia; 4 RSPCA, Wacol, QLD 4076, Australia

**Keywords:** dog, shelter, RSPCA, Queensland, admission source, outcomes, characteristics, stray, surrendered, adopted, euthanized, microchip, identification, breed, desexed

## Abstract

**Simple Summary:**

An up-to-date and comprehensive understanding of the characteristics and outcomes of dogs entering shelters is required for implementing targeted strategies to reduce euthanasia of healthy and treatable dogs in Australia. Currently, there are few up-to-date Australian data published on dogs entering shelters, and their outcomes. Of dogs entering the Royal Society for Prevention of Cruelty to Animals, Queensland shelters in 2014, the majority (58%) were strays and 26% were puppies. Only 18% of dogs >6 months were desexed. Most dogs were reclaimed (32%) or adopted (43%). Strategies targeted to locations and breeds overrepresented by admissions are required to reduce shelter admissions, particularly of strays and unwanted litters.

**Abstract:**

Over 200,000 stray and surrendered dogs are admitted to shelters and municipal facilities in Australia each year, and approximately 20% are euthanized. Contemporary, comprehensive data on the characteristics and outcomes of dogs entering shelters are required to reduce shelter admissions and euthanasia. However, there are currently limited up-to-date data published on dog admission into shelters. A retrospective single cohort study was conducted to describe the characteristics and outcomes of the dog population entering Royal Society for Prevention of Cruelty to Animals, Queensland (RSPCA-QLD) shelters in 2014 (*n* = 11,967). The majority of dog admissions were strays from the public (24%) or from municipal councils (34%). Just over a quarter of admissions were puppies, 18% of adults (>6 months) were desexed, and the majority of admissions were crossbred dogs (92%). The majority of owner surrenders (86%) were due to human-related reasons. Most dogs were reclaimed (32%) or adopted (43%) and aggression was the most common reason for euthanasia of adult dogs (45%). Low-cost or free desexing and identification programs targeted to areas and breeds contributing to high intake, and increased support services for owners at risk of surrendering their dog, should be trialed to determine their cost effectiveness in reducing shelter admissions and euthanasia.

## 1. Introduction

Over 200,000 stray and surrendered dogs are admitted to animal welfare shelters and local government animal facilities in Australia annually (9.3 dog admissions per 1000 residents), and 20% are euthanized [1]. Approximately 38% of Australian households own a dog, and dogs constitute 36% of all animals received by the largest animal welfare organization in Australia, the Royal Society of Prevention of Cruelty to Animals (RSPCA) [2,3]. Almost 46,000 dogs were admitted to RSPCA animal shelters throughout Australia during the 2013/2014 financial year, which overlapped with data collection for this project (calendar year 2014). Of these, 36% were reclaimed, 37% were rehomed and almost 16% were euthanized [3]. Furthermore, over 27% of the 7300 dogs euthanized by the RSPCA in 2013/2014 were in RSPCA Queensland (RSPCA-QLD) shelters, which admitted 15,000 dogs during this period [3]. This represents 3.2 dogs admitted annually per 1000 residents in Queensland, which is almost double the 1.9 dogs admitted per 1000 residents nationally to RSPCA shelters [3,4]. The financial cost of this situation is evident. In the 2013/2014 financial year, RSPCA Queensland spent over $15 million on its shelters, a $1.7 million increase from the previous year due to an increase in sheltered animals [5,6].

In addition to the financial costs, euthanasia of animals has detrimental effects on the psychological welfare of shelter workers [7,8,9,10,11]. Workers who are involved with euthanasia report experiencing stress, guilt and moral conflicts, increased levels of work-related stress, increased risk of substance abuse, and health problems [7,8,9,10,11]. Furthermore, shelter animals are also subjected to significant stresses, leading to illness, impaired psychological welfare and behavioral problems [12,13,14,15]. This can reduce the likelihood of successful adoption and increase the risk of euthanasia [12,13,14,15].

Despite the significance of these issues, there is little current information available on the profile of dogs entering shelters in Australia, the reasons and their outcomes. The most recent research which profiled dogs entering Australian shelters was published over ten years ago (2004) and focused on three Melbourne shelters [13]. It found that dogs in shelters were typically adult, small breed, stray males that were sexually entire, but there were considerable differences between each of the shelters [13,16]. Recently, a study profiling cats entering Australian RSPCA shelters identified risk factors for admission and euthanasia, and, based on this data, made recommendations for strategies to reduce the number of stray and surrendered cats in Australia [17]. This study demonstrated the value of comprehensive, up-to-date data on the population of cats in RSPCA shelters for guiding strategies to reduce shelter admissions and improve live release rates. A similar approach is warranted to better understand dog admissions and outcomes in shelters, so that more effective management strategies can be implemented. This is important because reducing shelter intake and increasing the number released alive, reduces the number of dogs euthanized [18,19].

The aims of this study were to describe the characteristics and outcomes of the dog population entering RSPCA-QLD shelters.

## 2. Experimental Section

### 2.1. Data Collection and Study Design

Data were sourced from the RSPCA’s Sheltermate© database and a retrospective single cohort study of all dogs entering Queensland RSPCA shelters in 2014 was conducted. Data obtained encompassed all first admissions of dogs from 1 January 2014 to 31 December 2014 into nine RSPCA-QLD shelters, and their associated outcomes from the nine shelters, adoption centers and associated pet shops. RSPCA-QLD operates four adoption centers where RSPCA animals can be adopted, but the full services of a shelter are not offered. The RSPCA also has contracts with selected pet shops to stock only RSPCA sheltered dogs and cats. Of the nine shelters, six had municipal council contracts and three did not accept strays from the general public (except under exceptional circumstances). Councils are local government bodies (similar to US counties) and are responsible for animal control and pound management. Pounds are council run facilities where stray, seized or surrendered dogs are kept for a set period of time until an owner comes forward or a dispute is resolved (e.g., licensing). If the dog remains unclaimed, it is transferred to a shelter facility (such as the RSPCA), rehomed or euthanized. Shelters are operated by welfare groups, and may or may not accept stray dogs, depending on government by-laws and shelter policies. Details of dogs accepted by the shelters were entered into the Sheltermate© system. All dogs were scanned for a microchip on admission, and most were assessed by a veterinarian within 24 to 48 h of admission.

Raw data were imported from the RSPCA’s Sheltermate© program and the Australian Bureau of Statistics (ABS) Census 2011 population data, and were manipulated in Microsoft Excel. Further clarification on definitions of data categories and shelter procedures was obtained through verbal and email communication with RSPCA-QLD staff. Data categories obtained included RSPCA allocated animal identification number, primary breed, estimated mature size (small/medium/large/extra large), crossbred status (crossbred/purebred), source of admission, postcode of source, date of entry, date of birth, age group on admission (puppy/juvenile/young adult/mature adult/senior), reason for surrender (if applicable), sex, previously desexed (yes/no), outcome, date of outcome, reason for euthanasia (if applicable), and human population by postcode. For dogs with multiple admissions in 2014 (identified by their allocated shelter identification number), only data for their first admission were used in the analysis. For the purpose of this study, the terms “dog” and “dogs” refer to dogs collectively, regardless of age. Puppies are dogs ≤6 months of age, whilst adults (>6 months) include juveniles (>6 months to 12 months), young adults (>12 months to 2 years), mature adults (>2 years to seven years) and senior adults (>7 years). Allocation to age category was based on their estimated birth date, and where this was not recorded, the age category allocated by the RSPCA was used. If no history of desexing status was obtained on admission, desex status was allocated based on external signs of sterilization (e.g., ear tattoo, abdominal scar or absence of testes). It was assumed that all dogs not listed as desexed prior to admission, or did not have a desex date prior to their admission date were entire. All dogs made available for adoption were desexed, and if it was noted during the procedure that there was evidence of prior desexing, this was updated in the database. Dog size was allocated by comparing a dog’s estimated mature height to a person of average height. Small dogs were defined as being less than knee height, medium dogs were approximately knee height, large dogs were thigh height and extra large dogs were approximately hip height.

For the purposes of this study, the sources of admission for each dog were organized under seven categories: owner surrender, stray, council admission, offspring of sheltered animals, euthanasia request, humane officer admission (employees of the RSPCA tasked with rescuing animals from situations where their welfare may be compromised), and other (Supplementary Materials, Table S1). Council admissions were mostly stray and some owner-surrendered dogs received by municipal councils and transferred to the RSPCA after a holding period of three to five days, or immediately under a pound management agreement (owner surrenders were transferred within 24 h). Surrender reasons were organized under human or dog related reasons, and a series of categories and subcategories (Supplementary Materials, Table S2). The outcomes for the dogs entering RSPCA shelters were allocated into eight categories: reclaimed, adopted, euthanized, in shelter, in foster, unassisted death, transferred out and other (Supplementary Materials, Table S3). Off-site euthanasias and adoptions recorded by the RSPCA (for example, adoptions through pet shops) were included in the data. Dogs with a date for outcome in 2015 were assumed to be “in shelter” on the conclusion of 2014, unless their outcome was “in foster”, whereby they were assumed to have already been “in foster” at the end of 2014. The RSPCA allocated reasons for euthanasia were organized into six categories, and a series of subcategories (Supplementary Materials, Table S4), for the purposes of this study.

Postcodes were recorded for where the animal was presented from (“lost/found postcode”), and for the person presenting the animal (“person postcode”) (i.e., dogs which were presented as strays may have been found in a different postcode to the address of the finder). For the purposes of this study the “lost/found postcode” was used. If no “lost/found postcode” was recorded, the “person postcode” was used. No postcode was recorded for 348 dogs, and these animals were excluded from the postcode analysis. Intake per 1000 residents within a postcode was calculated using data from the ABS 2011 Census. The Socio-Economic Index For Areas (SEIFA Rank) was used to identify the relative socio-economic status for all postcodes from which two or more dogs were received [20]. The index is a relative measure. A low score indicates relatively greater disadvantage and a lack of advantage in the area, and a high score indicates a relative lack of disadvantage and greater advantage in the area.

Data excluded from analysis included repeat admissions (*n* = 1212), entries with missing age data (*n* = 770), dogs deceased prior to arrival (*n* = 82) and duplicate or erroneous data (*n* = 4). A total of 11,967 initial dog admissions were used for the analysis. For each analysis, entries with incomplete data relating to the categories studied were excluded.

### 2.2. Statistical Analysis

Most results were descriptive statistics but the null hypothesis that the proportions of annual admission numbers are the same in each month was statistically tested using the Describe (version 3.02) module in WinPepi version 11.50 [21]. To determine whether the proportions of annual adult admissions that occurred in each month differed from an equal proportion admitted each month, overall goodness-of-fit was assessed using Pearson’s chi-square goodness-of-fit test [22]. For the proportions of annual admissions that occurred in each month, 95% confidence intervals were estimated [23]. Chi-square goodness-of-fit tests were also used to compare the observed frequency in each month with the combined observed frequencies of the other categories [24] to determine which months had significantly higher or lower proportions admitted. Sidak-corrected *p*-values were used. The same analyses were performed for puppy admissions. For all analyses, values of *p* < 0.05 were considered significant.

## 3. Results

### 3.1. Source of Admission

Of the dogs included in the analysis (11,967) that entered RSPCA Queensland shelters in 2014, most were strays presented to the RSPCA by members of the public (24%), or were council admissions (34%) (Table 1). Just under 20% of all dogs admitted were surrendered by owners directly to the RSPCA. The majority of adult admissions were council admissions (39%), whereas the majority of puppy (≤6 months) admissions were owner surrenders (32%). A similar proportion of adults and puppies were admitted as strays.

### 3.2. Age, Sex and Desex Status

**Figure 1:**
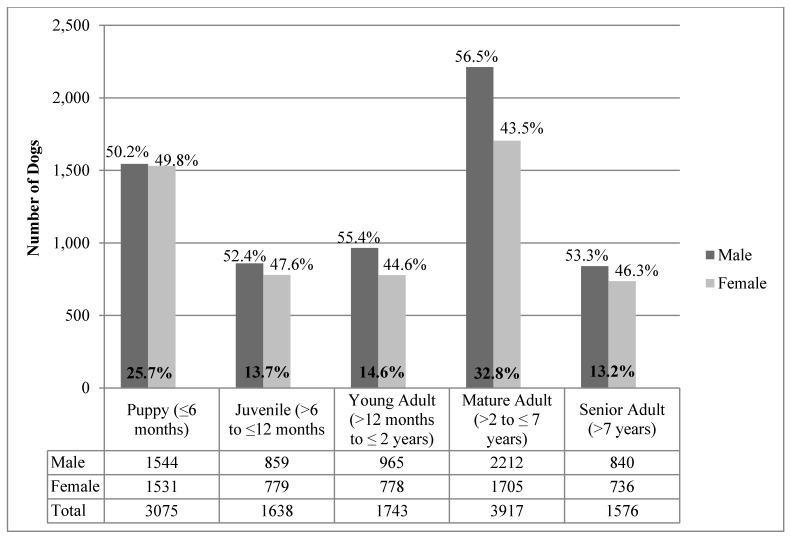
Summary of age groups and sex for 11,949 dogs admitted to RSPCA Queensland shelters in 2014 (18 dogs were excluded from the analysis because sex was not recorded).

The majority (74%, 8874) of dog admissions were adults older than six months and 26% were puppies ≤6 months of age (Figure 1). The biggest age category (33% of admissions) were mature adults >2 years to seven years. Similar numbers of juveniles (7–12 months), young adults (13–24 months) and senior adults (>7 years) were admitted. More males (54%) were admitted than females (46%) and there were 10% more adult males than adult females. In puppies, there was minimal difference between males than females (0.4%). The difference in the ratio of males to females increased as dog age increased up to seven years with a 13% difference between mature adult males and females (Figure 1).

More adult males were admitted as council admissions (41%) than adult females (37%), whereas, for other admission sources, the ratio of adult males to adult females was very similar (Table 1). For puppies for all sources of admissions, the ratio of males to females was similar. Only 14% of dogs were listed as desexed on entry, and the remaining 86% were presumed to be entire. Just 2% of puppies were known to be desexed on admission, whilst 18% of adult dogs were known to be desexed on admission. Similar ratios of males and females were known to be desexed prior to arrival, with a slightly higher percentage of females desexed (Table 2). The same proportion of male and female puppies were desexed. The proportion of dogs desexed prior to admission increased as age increased (Table 2) with the exception of senior adults (15%). One quarter of mature adults (two to seven years) were desexed prior to entry. The admission group with the greatest proportion of desexed animals was owner surrenders (21%).

### 3.3. Postcode of Source

When shelter intakes were compared to the human population of the “lost/found” postcode, the five greatest intakes per 1000 residents occurred in Kingaroy, Caboolture, Gympie, Morayfield and Burdell, and their intakes ranged from 15–21/1000 residents (intakes of less than 50 dogs per postcode were excluded from this analysis) (Table 3). However, the five greatest total intakes were from Toowoomba, Caboolture, Gympie, Townsville and Dakabin. Two of the top five total dog admissions postcodes were also within the top five intakes per 1000 residents (4510 and 4570), whereas the region with the greatest number of admissions only had an intake of eight dogs per 1000 residents. Suburbs and towns included within each postcode are recorded in the Supplementary Materials (Table S5). The Socio-Economic Index For Areas (SEIFA) Rank for postcodes receiving two or more dogs ranged from 419 to 5 (median index 242, lower quartile 126). Of the five postcodes with the highest intakes per 1000 residents and total intakes of at least 50 dogs, three of the five had scores of around or below the lower quartile (median index 94), indicating relatively greater disadvantage and a lack of advantage in the area. Notably, the 17 postcodes with scores of >400, all but two had intakes of <1 dog/1000 residents and the other two had intakes of 1.4 and 2.1 dogs/1000 residents.

### 3.4. Size & Primary Breed

**Figure 2:**
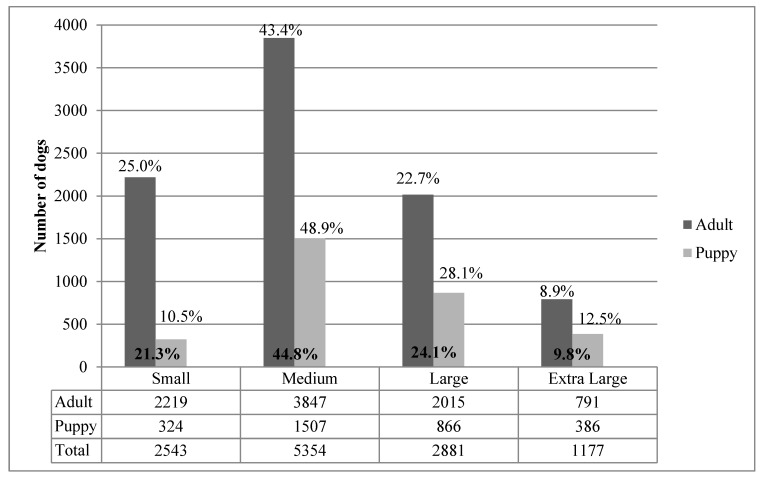
Estimated adult size (dog size was defined as follows: small dogs are less than average knee height, medium dogs are approximately knee height, large dogs are approximately thigh height and extra large dogs are hip height or taller) of 11,955 dogs admitted to RSPCA-QLD shelters in 2014 (12 dogs were excluded from the analysis because estimated mature size was not recorded).

When grouped by size, most (45%) were medium-sized, with similar proportions of large breed (24%) and small breed (21%), and only 10% were classed as extra large dogs (Figure 2). A greater percentage of adult dogs were small breed (25%) than puppies (10%), and a greater percentage of puppies were medium, large or extra large breed than adults.

The vast majority of dogs admitted were cross breeds, with just 8% of dogs listed as pure breeds. Of all dogs admitted, 83% were crossbreed or purebred dogs of 20 breeds (Table 4). The Staffordshire Bull Terrier was the most prevalent primary breed for both purebred (27%) and crossbred (19%) dogs, and was the primary breed for 20% of all dogs admitted (cross and pure breed). The five most common primary breeds for adults admitted were Staffordshire Bull Terrier, Australian Cattle Dog, Maltese Terrier, Labrador Retriever and Kelpie. The five most common breeds for puppies admitted were Staffordshire Bull Terrier, Bull Arab, Australian Cattle Dog, Kelpie and Border Collie.

### 3.5. Date of Admission

**Figure 3:**
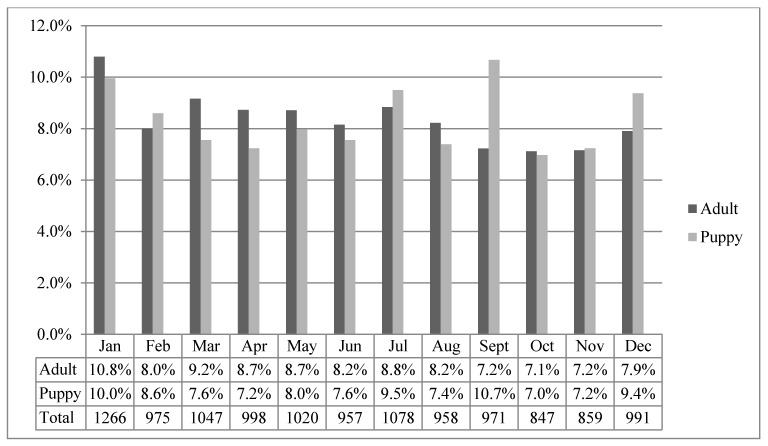
Numbers of admissions to RSPCA-QLD shelters in 2014 by month, and percentages of annual admissions by month within each of adult dogs (*n* = 8883) and puppies (*n* = 3084).

For the year 2014, within each of adults and puppies, the proportions of annual numbers of admissions differed by month (*p* < 0.001 for each). A significantly higher than expected proportion of the year’s adult admissions occurred in January (10.8%; 95% CI 10.2–11.5%; *p* < 0.001) and lower than expected proportions occurred in September (7.2%; 95% CI 6.7–7.8%; *p* = 0.002), October (7.1%; 95% CI 6.6–7.7%; *p* < 0.001) and November (7.2%; 95% CI 6.6–7.7%; *p* = 0.001). A significantly higher than expected proportion of the year’s puppy admissions occurred in January (10.0%; 95% CI 8.9–11.1%; *p* = 0.015) and September (10.7%; 95% CI 9.6–11.8%; *p* < 0.001) (Figure 3 and Table 5).

**Figure 4:**
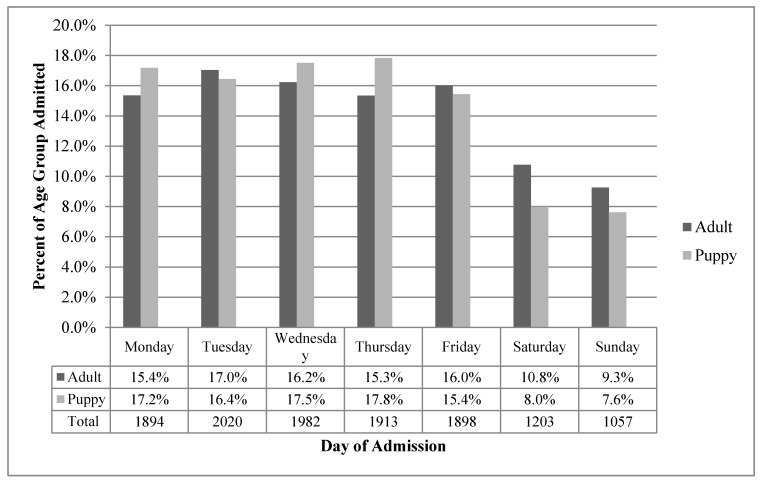
Summary of admission group and day of entry of 11,967 dogs processed through RSPCA-QLD shelters in 2014.

Fewer dogs were admitted on weekends than on week days, with only 20% of adults and 16% of puppies admitted on weekends (Figure 4). Adult admissions remained approximately equal from Monday to Friday, with 15% to 17% of admissions occurring each day. A similar proportion of total puppy admissions (84%) occurred on weekdays as adult admissions (80%).

### 3.6. Reason for Surrender

Of the 2752 dogs admitted as owner surrenders or euthanasia requests, 68% had a reason for surrender recorded. The majority of these (86%) were admitted for human-related reasons, and 14% were admitted for dog-related reasons (Table 6). Most surrendered dogs (40%) were classified as unwanted pets. Puppies contributed to the majority of these admissions, with 69% of puppy admissions and just 20% of adults surrendered because they were unwanted. The majority of unwanted adult dogs were surrendered because their owners felt they made a poor decision (41%), whereas the majority of unwanted puppies were surrendered because too many dogs were owned (81%) (this category included unwanted litters). The majority of adult dog surrenders were due to a change in circumstances (29%), the most frequent of which was the owners moving or travelling (60%). The most common dog-related reason for surrender was behavioral (13%) with 35% (*n* = 88) of these surrendered for aggressive or predatory behavior. Sixteen percent of all surrenders were due to owners being unable to afford care or treatment.

### 3.7. Outcomes

The majority of dogs admitted into RSPCA QLD shelters in 2014 were adopted (43%), and 32% of dogs were reclaimed. Just 14% of dogs were euthanized and 7% were still residing in the shelter or in foster care at the conclusion of 2014 (Table 7). The most common outcome for puppies was adoption (71%), whereas adults were most frequently reclaimed (39%). Just 10% of puppies were reclaimed. A greater percentage of adults were euthanized than puppies (17% of adults and 5% of puppies). Adoption was the second most frequent outcome for adult dogs, with 34% of all adults adopted, 55% of unclaimed adults adopted and 63% of surrendered adults adopted. Of the 68% of dogs that were not reclaimed, 79% of puppies were adopted and just 5% of puppies were euthanized, whereas 27% of unclaimed adults were euthanized.

A reason for euthanasia was recorded for all 1619 dogs euthanized in RSPCA-QLD shelters in 2014. More than half (53%) of all euthanasias were for behavioral reasons with 80% of these euthanized for aggressive behavior (Table 8). Approximately 45% of adult euthanasias were for aggressive behavior, compared to just 15% of puppy euthanasias. Most puppy euthanasias were based on health (67%), and 45% of these were euthanized for parvovirus infection or contact with a parvovirus infected animal. Owner requested euthanasia was the second most common reason for adult euthanasia (21%), but a similar proportion of adult euthanasias were due to health (19%) (most commonly musculoskeletal or neoplastic diseases). Just 5% of puppy euthanasias occurred as a result of an owner’s request.

## 4. Discussion

### 4.1. Source of Admission

The concept of capacity for care is based on the premise that animal rescue organizations have a finite number of animals for which they can provide an acceptable level of care [25,26,27]. If intake exceeds this number, shelters may have to euthanize potentially adoptable animals to free up space. By reducing avoidable admissions and improving live release rates, shelters are better able to care for, and ensure positive outcomes for the animals in their care, which in turn reduces the number of admitted animals euthanized [28]. To reduce intake, communities need to find strategies to prevent avoidable admissions [27].

#### 4.1.1. Strays and Council Admissions

The majority of dogs entering RSPCA shelters in 2014 were council admissions (34%), most of which are stray dogs rather than owner surrendered animals [29]. Combined with the 24% of strays surrendered by members of the public, this implies that almost 60% of the dogs admitted into RSPCA-QLD shelters were strays. This is similar to a New Zealand study from 1999 to 2006 (52% stray admissions) [30], but less than in a Victorian study from 2004 (83.8% stray admissions) [13], and greater than in a UK study conducted in 2010 (25.8% stray admissions) [14]. These differences likely reflect regional differences in containment of dogs, and admission policies between shelters. The differences also reflect how animals are classified on admission. Most council admissions were assumed to be strays, however, to more accurately estimate the magnitude of the stray population in our study, it is recommended that source of council admissions should also be recorded.

With strays and council admissions contributing to an overwhelming majority of dogs entering RSPCA-QLD shelters, strategies to prevent dogs from straying and increase their reclaim rates need to be considered. A recent study (2015) investigating the microchip data of dogs entering RSPCA-QLD shelters found that, despite mandatory microchipping of dogs in Queensland, just 28% of stray dogs admitted into the shelters were microchipped, and, of those, 37% had inaccurate details of the owner or were not registered with any database [31]. Reclaim rates were significantly higher for dogs with no microchip data problems (87% reclaimed), whereas there was increased length of stay in shelters, and decreased reclaim rates for dogs with microchip information problems (69% reclaimed), or an absence of a microchip (37% reclaimed) [31].

Reducing shelter intake is closely associated with reduced euthanasia of animals in the shelter [18,19]. An increase in both the percentage of microchipped dogs, and proportion with current owner contact details registered on a database, are required to reduce the numbers of stray and council admissions entering RSPCA shelters, and increase reclaim rates. This may include microchip database companies establishing a reminder system for owners to update contact details (SMS texts, emails, and letters), microchip awareness campaigns and discount microchipping targeted to locations overrepresented by stray admissions.

However, inherent limitations of the microchipping system are that finders of stray dogs are unable to access ownership information without a microchip reader. Visual identification with a tag displaying the owner’s contact details facilitates the general public returning stray animals without requiring the assistance of organizations such as shelters, councils and veterinary practices. Research is warranted to determine if provision of free engraved ID tags is cost effective for municipalities and welfare agencies to reduce stray admissions. A centralized database for lost and found animals may also aid the general public, shelters and councils in reuniting stray animals with their owners.

The number of dogs presented to shelters as strays could also be reduced by changing the focus of animal control officers from impounding stray animals, to working with communities to prevent dogs from straying, and increasing compliance with registration (dog licensing) and identification [32]. Although it is often prohibited by municipal by-laws, some municipalities permit animal control officers to directly return stray dogs with identification to the owner, even if dog registration is not current [29]. This reduces stray admissions and is likely more cost effective for municipalities, given that some impounded dogs are not reclaimed because of cost [1,33]. Increasing the cost to municipal councils per dog managed by the RSPCA (and other animal welfare agencies with council contracts) may provide an incentive for municipalities to engage more proactively in increasing the number of dogs that can be returned directly to the owner.

#### 4.1.2. Owner Surrenders and Euthanasia Requests

The proportion of owner surrenders and euthanasia requests admitted to RSPCA-QLD (23%) was less than reported in a UK study (56%) and a New Zealand study (47%) [14,30]. Most (19%) of these RSPCA-QLD admissions were owner surrenders and only 4% were euthanasia requests, of which most were adult dogs. However, almost a third of puppy admissions were owner surrenders, which is similar to a Victorian study which found that 28% of puppies were admitted as owner surrenders [13]. Likewise, it was found that 15% of adult admissions were owner surrenders, compared to 14% reported in the Victorian study [13].

Interviews with owners who relinquished pets revealed that most owners are reluctant to surrender their pets, often prolonging the decision and opting for surrender as a last resort [34,35]. Many shelter organizations (including the RSPCA) offer resources aimed at assisting owners with their pet’s problems. In particular, RSPCA-QLD require people surrendering their pets to attend an appointment prior to surrender, which provides an opportunity for shelter staff to explore reasons for surrender and provide advice, and gives owners time to reconsider their decision [29]. However, many owners are unaware of the availability of these resources [36]. Furthermore, they will often spend a considerable amount of time coming to a decision to surrender, and consequently, these efforts made at the point of surrender may have a reduced effect [34]. Increasing public awareness of the availability of these services earlier in the decision making process may aid in reducing admissions. For example, Pet Help Partners in New York implemented a phone-based support service where owners faced with surrendering their pets were provided with counseling and advice on finding alternatives to surrender [35]. It was found that retention rates were high for owners who contacted the organization prior to the point of surrender [35]. A similar approach would likely be effective in Australia, and pet retention strategies should be considered by municipalities as a service to pet owners, and likely represents better use of taxpayers’ money than accepting all surrenders.

### 4.2. Age, Sex and Desex Status

Over a quarter (26%) of admissions to RSPCA-QLD shelters were puppies, which is greater than the 10% reported by the Victorian study. Furthermore, just 18% of adult dogs entering RSPCA-QLD shelters were desexed, slightly less than reported in the 2004 Victorian study (23%) [13], and considerably less than the average of 78% of owned dogs in Australia [2,37]. The proportion of dogs desexed prior to arrival increased as age increased (with the exception of senior dogs), however dogs reach puberty at an average age of six to nine months [38], so most dogs entering RSPCA-QLD shelters were desexed too late to prevent breeding. This indicates that a significant contributor to shelter admissions was likely to be poor desexing compliance, leading to excess puppies.

Studies have found significant declines in shelter intakes of cats occurred when subsidized desexing or trap neuter return programs were implemented [39,40], but the effectiveness of subsidized desexing programs has had a variable impact on shelter intake of dogs. A study conducted in 2011 in San Jose, California identified Chihuahuas as a significant contributor to shelter intake, and implemented a free desexing program targeted at Chihuahuas and Chihuahua crosses [40]. As a result, overall dog admissions decreased from seven dogs per 1000 humans to 6.1 in two years [40]. Similarly, a free desexing program in Austin, Texas targeted to zip codes with high shelter intake, low average income and scarcity of veterinary facilities slowed the increase in dog admissions, compared to intakes from other zip codes [39]. However, a subsidized desexing program primarily involving adopted shelter animals (which had not yet been sterilized) and low income families in New Hampshire did not significantly reduce intake or euthanasia of dogs, whilst cat intake and euthanasia declined significantly [39]. These findings suggest that subsidized desexing programs may be more effective for reducing shelter intakes of cats than for dogs (reflecting the higher proportion of incoming cats less than six months of age compared to dogs [17]) and subsidized desexing programs for dogs should be targeted for locations and breeds contributing to highest intakes.

### 4.3. Postcode of Source

The greatest overall number of dog admissions primarily occurred in large regional centers, and 12 of the top 20 postcodes all had admissions of ≥10 dogs/1000 residents, which is above the average for Australia (9.3 dogs/1000 residents) [1]. A 2010 Australian study [41] found a significant relationship between owner demographics and pet confinement, and household income and obedience training attendance. A US study [42] found that owners relinquishing their animals were more likely to be less than 50 years old, and not have complete education beyond high school. Furthermore, in underserved neighborhoods within the USA (which are typically low socio-economic areas), approximately 87% of pets were found to be entire, compared to the national average of about 9% [33]. When people in underserved communities were provided with the access to spay/neuter resources, including cost assistance and transportation, the percentage of desexed pets in the community increased to national levels [33]. Socio-economic position, lifestyle and demographics play a role in pet relinquishment and straying [18]. This is consistent with our findings where three of the five postcodes with the highest intakes per 1000 residents had SEIFA ranks around or below the lower quartile, indicating relatively greater disadvantage and a lack of advantage in the area.

Desexing and identification programs, as well as programs which assist people to keep their pet, that are targeted to areas with a combination of high intake per capita and high total intake, are likely to have greatest effect in reducing intake and subsequent euthanasia. Many of these locations will be relatively disadvantaged, and require financial and other assistance to increase desexing and identification rates, and for owners to keep their pet rather than surrender it.

### 4.4. Primary Breed and Mature Size

Of the 69,274 puppies registered by the Australian National Kennel Council (ANKC) in 2014, Staffordshire Bull Terriers (10%), Labrador Retrievers (7%), German Shepherds (5.2%), Golden Retrievers (4.2%) and Border Collies (3.9%) were the top five breeds registered [43]. Similarly, Staffordshire Bull Terriers (20%) and Border Collies (5%) were among the five most prevalent breeds admitted to RSPCA-QLD shelters. Three of the five most prevalent primary breeds (Australian Cattle Dog, Kelpie and Border Collie) admitted into RSPCA-QLD shelters are classified as working dogs by the ANKC [43]. High intelligence and energy levels may contribute to a greater likelihood for these breeds to stray or have behavioral problems related to boredom, and be admitted into shelters [44].

According to a 2013 Australian pet ownership report, 50% of owned dogs were crossbreds [37]. However, assuming that most planned litters are purebreds, the high prevalence of crossbred puppies (98%) that entered RSPCA-QLD shelters suggests that there are still a significant number of unplanned litters occurring in the community. Additionally, a US study [42] found that crossbreds were at a greater risk of relinquishment. A free desexing program targeting the most prevalent breed entering shelters in San Jose, California resulted in an overall decrease in shelter admissions [40]. A similarly targeted subsidized desexing program focusing on Staffordshire Bull Terriers and their mixes (20% of admissions) in postcodes overrepresented by admissions may reduce dog admissions, and be cost-effective.

### 4.5. Date of Admission

Dogs generally have two estrus cycles a year, which commonly occur in Spring and Autumn [38]. Pregnancy lasts for two months and puppies are weaned from approximately six weeks old (i.e., summer to early autumn and winter to early spring) [38]. However, previous studies have indicated that there is little to no seasonal pattern in dog admissions, compared to the significant seasonal patterns noted in cat admissions [30,39,45]. Despite this, variations in puppy admissions were noted in our study, with significantly greater proportions admitted in September (Spring) and January (Summer).

A study of Victorian shelter admissions found an increase in total admission numbers in January and December, and suggested that this may be due to straying during thunderstorms or changes in human activity (e.g., school holidays). Our study also found that a significantly higher proportion of adult and puppies were admitted in January. Considering that the majority of adult and puppy admissions (63% and 45%, respectively) were strays or council admissions, thunderstorm activity and New Year’s fireworks are possible explanations for our findings. Furthermore, the 2014 Queensland school holidays occurred in January, April, July, September/October and December [46]. This partially corresponds to the January and September peaks in puppy admissions, although it was noted that a significantly lower proportion of adult admissions occurred in September, October and November. In order to understand these admissions variations, further study into the reasons associated with admissions is required. A limitation of our study is the use of data from a single year. It is uncertain if these variances in monthly admission are repeatable over a number of years.

### 4.6. Reason for Surrender

The most frequently cited reasons for surrender included dog behavior (especially aggression and escaping), accommodation problems, and owner health and personal issues [13,34,47]. Similarly, our study found that adult dogs were most commonly surrendered due to changes in owner's circumstances (29%), behavioral problems (22%) and as unwanted animals (20%), whereas puppies were most commonly surrendered as unwanted animals (69%) (most due to overpopulation), and due to financial constraints (20%).

Despite puppies contributing to 26% of the population of admitted dogs, they contributed to 41% of the surrendered population. This supports previous findings that risk of relinquishment decreases with length of ownership and age [42]. It is likely that the majority of the 56% of puppies surrendered due to “too many animals” were the result of unwanted litters, especially considering that 98% of puppies were crossbred. However, the second most frequent reason for surrender of puppies was financial considerations (20%). It may be that some of these puppies were acquired impulsively, or at no or low cost. It has previously been reported that a lower level of education, and erroneous beliefs about desexing dogs and their reproductive cycles lead to a greater risk of relinquishment [42,47]. The positive link between low socio-economic status and dog admissions suggests that providing assistance with transport and access to low-cost and free desexing targeted to these underserviced areas is warranted [30]. A 2004 study of Victorian shelters found that 40% of owner-related reasons for surrender were due to accommodation or moving [13], whereas 20% of owner-related admissions in our study were due to moving or travelling, or accommodation problems. A US study into reasons for relinquishment found that 40% of owners who relinquished their animals for the reason of “moving” did so due to landlord conditions [48]. Strategies to change attitudes of landlords, body corporates and real-estate agents to pets, and change legislation regarding pets in rental properties are needed to reduce the number of pets surrendered. At the time of writing, several Australian states (including Queensland) were reviewing their Residential Tenancies Acts, and submissions have been made advocating the “no pets” clause should be deemed illegal [49].

A limitation of our study was that a single reason for surrender was recorded, whilst owner surrenders are often multifactorial [34,48]. To gain a greater understanding for the motivations behind owner relinquishment, shelters should consider providing owners with the option to provide more than one reason for surrender. A 2000 US study examining risk factors for relinquishment found that behaviors exhibited by relinquished animals were not unique to them, and were also exhibited by owned animals [42]. Additionally, another US study found that 29.5% of surrendered dogs were surrendered for non-aggressive behavioral reasons [47]. It is suggested that the risk of relinquishment depends on the owner’s tolerance of these behaviors, expectations and knowledge [42], and a dog’s perceived behavior is dependent on owner’s prior experience [50]. More than 52% of owners relinquishing their animals for health and personal reasons believed that animals misbehaved out of spite [47]. Therefore, a proportion of the 22% of adult dogs admitted to RPCA-QLD shelters for behavioral reasons were likely due to a mismatch of the dog’s behavior and the owner’s expectations. Greater public accessibility to low-cost assistance with behavior problems, and greater awareness of RSPCA-QLD services for owners challenged with dog behavior problems are required to reduce behavior-related surrenders.

### 4.7. Outcomes and Reasons for Euthanasia

Fewer dogs were euthanized in our study (14%) and a much greater proportion adopted (43%) compared to a 2008 New Zealand study (36% adopted and 45% euthanized) [30], and a 2004 Victorian study (21% adopted and 32% euthanized) [13]. Our study found that the majority of euthanized dogs were euthanized for aggression (42%), and 23% for health reasons. In contrast, the Victorian study found that, of the dogs that were euthanized, 34% were due to health problems and 24% were due to aggression [13]. The greater proportion of adoptions in our study may be a result of the RSPCA’s high profile adoption campaigns and marketing strategies, and initiatives to reduce intake, such as, surrender by appointment and their Animal Training and Behavior Centre. In Victoria, the majority of owners acquired their pets through breeders (50%), pet shops (11%) and friends and family (11%) with just 14% of pets acquired from shelters [50]. To encourage the public to purchase animals through a shelter there needs to be greater public awareness of the quality of animals available for adoption, which are behavior and health checked, desexed, vaccinated and microchipped, and unlike many other sources, can be returned after a trial period if necessary. Many of the human-related reasons for surrender (such as landlord or council restrictions) may also prevent suitable potential owners from adopting a dog. Revising tenancy agreement legislation relating to “no-pets” clauses may also aid in increasing adoption rates.

Future studies into the associations between dog characteristics (such as age, breed, sex and desex status), and their outcomes and reasons for euthanasia could be conducted using the results of our study. By understanding what dog-associated factors contribute to positive outcomes (reclaim or adoption), shelters such as the RSPCA can better target their programs to increase these outcomes.

### 4.8. Study Limitations

Our study only investigated dogs entering RSPCA-QLD shelters, the management of which may be different to other shelters within Queensland and Australia. Furthermore, there were limited data available on the characteristics of Queensland’s owned dog population. Therefore, comparisons could not be made in regards to the prevalence of characteristics of the sheltered population compared to the owned population. Inconsistencies occurred in the shelters’ data collection due to multiple people and shelters contributing to data entry. Furthermore, the RSPCA only records one reason for surrender and euthanasia, and potentially complex circumstances were not captured in the data. Research has shown that there is typically more than one reason associated with surrender [36,47,51]. There were also overlapping categories and the potential for differences in interpretation. For example, dogs that were surrendered because they were “acquired without consent of landlord/household” could potentially have been classified as “landlord won’t allow”. Definitions of dog age groups were not provided, and therefore varied depending on the personnel entering the data. Finally, variation exists between our results and previous studies, which may be due to date and geographical differences. This emphasizes the need for contemporary and targeted data to guide strategies to further reduce shelter admissions and euthanasia.

## 5. Conclusions

The proportion of desexed animals entering shelters is lower than for owned dogs in the general population, and puppy admissions contribute to a quarter of shelter admissions. Stray adult dogs and Staffordshire Bull Terrier crossbreds are major contributors to admissions. Admission numbers vary month to month with higher proportions of puppies and adults admitted in January. Further research is required to determine if variations are consistent over a number of years, and what they were associated with (e.g., breeding or changes in human activity). Puppies contribute to a considerable proportion of owner surrenders and the majority of surrenders are due to human-related factors, mostly too many animals or due to a change in circumstances. The most prevalent dog-related reasons for surrender are behavioral problems. The majority of dogs admitted are reclaimed or adopted, and aggressive behavior is the most common reason for euthanasia. Based on our study findings, the cost-effectiveness of programs to reduce intake and euthanasia, and increase reclaim or adoption rates should be evaluated. These could include low-cost or free desexing and identification programs targeted to breeds and locations associated with high total intake and intake per capita. Increased availability of support services to assist owners challenged with situations that increase the risk of surrender, such as problem behaviors, lack of pet-friendly accommodation, and other personal issues (including lack of finances for pet health care), will likely reduce admissions and subsequent euthanasia.

## Figures and Tables

**Table 1:** Summary of age group (adult > 6 months and puppy ≤ 6 months), sex and admission source of 11,949 dogs processed through RSPCA Queensland shelters in 2014.

Admission Source	All Dogs			Female		Male	
	Total	Adult	Puppy	Adult	Puppy	Adult	Puppy
Council	34% (4087)	39% (3471)	20% (616)	37% (1483)	19% (297)	41% (1988)	21% (319)
Stray	24% (2910)	24% (2130)	25% (780)	24% (979)	24% (375)	24% (1151)	26% (405)
Owner Surrender	19% (2279)	15% (1310)	32% (969)	15% (602)	32% (488)	15% (708)	31% (481)
Humane Officer	6% (749)	6% (567)	6% (182)	7% (266)	5% (83)	6% (301)	6% (99)
Euthanasia Request	4% (469)	5% (456)	<1% (13)	5% (219)	<1% (7)	5% (237)	<1% (6)
Offspring	1% (128)	<1% (5)	4% (123)	<1% (4)	4% (64)	<1% (1)	4% (59)
Other	11% (1327)	11% (935)	13% (392)	11% (445)	14% (217)	10% (490)	11% (175)
Total	11,949	8874	3075	3998	1531	4876	1544

Eighteen dogs were excluded from the analysis because sex was not recorded.

**Table 2:** Summary of desex status for 11,967 dogs admitted to RSPCA Queensland shelters in 2014.

Admission Groups		Desexed
Adults	Female	19% (765)
	Male	18% (878)
	Total	18% (1643)
Puppies	Female	1% (17)
	Male	1% (24)
	Total	2% (41)
Puppy		1% (41)
Juvenile		10% (161)
Young Adult		21% (825)
Mature Adult		25% (394)
Senior Adult		15% (263)
Council		15% (603)
Owner Surrender		21% (482)
Stray		10% (294)
Other		15% (204)
Humane Officer		7% (52)
Euthanasia Request		10% (48)
Offspring		1% (1)
**Total**		**14% (1684)**

Eighteen dogs were excluded from the analysis because sex was not recorded.

**Table 3:** Shelter intake according to source postcode.

Twenty Greatest Intakes by Dog Admissions per 1000 Residents					Twenty Greatest Intakes by Total Dog Admissions				
Postcode	Population	Dog Admissions	Intake per 1000 Residents	SEIFA Rank	Postcode	Population	Dog Admissions	Intake per 1000 Residents	SEIFA Rank
4610	14,486	301	20.78	129	4350	102,244	819	8.01	207
4510	42,206	725	17.18	62	4510	42,206	725	17.18	62
4570	39,094	670	17.14	68	4570	39,094	670	17.14	68
4506	19,702	306	15.53	165	4814	44,389	506	11.40	287
4818	20,307	313	15.41	310	4503	34,305	418	12.18	262
4810	21,523	328	15.24	289	4670	77,159	365	4.73	82
4508	19,673	292	14.84	37	4810	21,523	328	15.24	289
4502	8499	117	13.77	313	4818	20,307	313	15.41	310
4815	20,523	275	13.40	169	4506	19,702	306	15.53	165
4021	9929	131	13.19	94	4610	14,486	301	20.78	129
4503	34,305	418	12.18	262	4508	19,673	292	14.84	37
4580	4513	52	11.52	25	4815	20,523	275	13.40	169
4814	44,389	506	11.40	287	4500	37,629	270	7.18	338
4501	5356	58	10.83	125	4817	29,741	234	7.87	309
4504	16,224	172	10.60	72	4300	45,133	203	4.50	232
4505	18,726	197	10.52	271	4505	18,726	197	10.52	271
4562	7193	67	9.31	330	4504	16,224	172	10.60	72
4019	19,007	164	8.63	110	4019	19,007	164	8.63	110
4076	6794	56	8.24	87	4680	50,831	154	3.03	303
4615	6859	56	8.16	27	4812	19,476	134	6.88	176

The Socio-Economic Index For Areas (SEIFA) rank is a relative measure of socio economic advantage and disadvantage. A low score indicates a relatively greater disadvantage and a lack of advantage, than a high score. Rank for postcodes receiving two or more dogs ranged from 419 to 5 (median index 242, lower quartile 126). Postcodes were not recorded for 371 dogs; these were excluded from the analysis.

**Table 4:** Twenty most prevalent breeds of 11,967 dogs processed through RSPCA Queensland shelters in 2014.

Primary Breed	Total			Adult		Puppy	
	Total	Pure Breed	Cross Breed	Pure Breed	Cross Breed	Pure Breed	Cross Breed
Staffordshire Bull Terrier	20% (2342)	27% (255)	19% (2087)	27% (243)	20% (1592)	26% (12)	16% (495)
Australian Cattle Dog	8% (968)	2% (22)	9% (946)	2% (20)	7% (577)	4% (2)	12% (369)
Kelpie	7% (825)	2% (18)	7% (807)	1% (13)	6% (491)	11% (5)	10% (316)
Bull Arab	7% (802)	<1% (1)	7% (801)	<1% (1)	5% (430)	0% (0)	12% (371)
Border Collie	5% (598)	6% (60)	5% (538)	7% (59)	5% (372)	2% (1)	5% (166)
Labrador Retriever	5% (560)	8% (79)	4% (481)	9% (78)	5% (363)	2% (1)	4% (118)
Maltese Terrier	4% (507)	2% (15)	4% (492)	1% (12)	6% (444)	7% (3)	2% (48)
Fox Terrier	4% (429)	2% (15)	4% (414)	2% (14)	5% (364)	2% (1)	2% (50)
Mastiff	3% (371)	<1% (1)	3% (370)	<1% (1)	3% (252)	0% (0)	4% (118)
Jack Russell Terrier	3% (350)	2% (21)	3% (329)	2% (21)	3% (273)	0% (0)	2% (56)
German Shepherd	3% (309)	4% (42)	2% (267)	4% (39)	2% (178)	7% (3)	3% (89)
Shih Tzu	2% (269)	1% (10)	2% (259)	1% (8)	3% (215)	4% (2)	1% (44)
Rottweiler	2% (259)	4% (35)	2% (224)	4% (32)	2% (170)	7% (3)	2% (54)
Chihuahua	2% (229)	1% (8)	2% (221)	1% (8)	2% (182)	0% (0)	1% (39)
Rhodesian Ridgeback	2% (227)	0% (0)	2% (227)	0% (0)	2% (140)	0% (0)	3% (87)
Great Dane	2% (197)	1% (5)	2% (192)	<1% (4)	2% (133)	2% (1)	2% (59)
Irish Wolfhound	2% (193)	0% (0)	2% (193)	0% (0)	2% (130)	0% (0)	2% (63)
Bullmastiff	1% (170)	<1% (3)	2% (167)	<1% (3)	1% (100)	0% (0)	2% (67)
Shar Pei	1% (167)	2% (17)	1% (150)	2% (16)	1% (89)	2% (1)	2% (61)
Boxer	1% (167)	2% (21)	1% (146)	2% (21)	1% (103)	0% (0)	1% (43)
Total Population (11,967)	100% (11,967)	8% (943)	92% (11,024)	7% (897)	67% (7986)	<1% (46)	25% (3038)

Percent of total population included in top 20 breeds = 83% (9939).

**Table 5:** Numbers and percentages of the annual admissions to RSPCA-QLD shelters by month in 2014 within each of adult dogs and puppies. *p*-values compare the observed frequency in each month with the combined observed frequencies of the other categories within columns.

Month	Adults			Puppies		
	Number (%)	95% CI	*p*-Value	Number (%)	95% CI	*p*-Value
January	959 (10.8)	10.2–11.5%	<0.001	307 (10.0)	8.9–11.1%	0.015
February	710 (8.0)	7.4–8.6%	0.970	265 (8.6)	7.6–9.7%	1.000
March	814 (9.2)	8.6–9.8%	0.058	233 (7.6)	6.7–8.6%	0.801
April	775 (8.7)	8.1–9.3%	0.919	223 (7.2)	6.4–8.2%	0.298
May	774 (8.7)	8.1–9.3%	0.933	246 (8.0)	7.1–9.0%	1.000
June	724 (8.2)	7.6–8.7%	1.000	233 (7.6)	6.7–8.6%	0.801
July	785 (8.8)	8.3–9.5%	0.675	293 (9.5)	8.5–10.6%	0.222
August	730 (8.2)	7.7–8.8%	1.000	228 (7.4)	6.5–8.4%	0.544
September	642 (7.2)	6.7–7.8%	0.002	329 (10.7)	9.6–11.8%	<0.001
October	632 (7.1)	6.6–7.7%	<0.001	215 (7.0)	6.1–7.9%	0.079
November	636 (7.2)	6.6–7.7%	0.001	223 (7.2)	6.4–8.2%	0.298
December	702 (7.9)	7.4–8.5%	0.852	289 (9.4)	8.4–10.5%	0.388

**Table 6:** Reasons for surrender organized according to human or dog related factors for 1876 dogs surrendered to RSPCA-QLD shelters in 2014 (a surrender reason was not recorded for 876 surrendered dogs, and therefore were not included in the analysis).

Human/Dog Related Factor	Surrender Reason Category	Surrender Reason Sub Category	Total	Adult	Puppy
**Human**			**86% (1615)**	**78% (860)**	**98% (755)**
	**Unwanted**	**Total-Unwanted**	**40% (751)**	**20% (221)**	**69% (530)**
	*Proportion of total surrendered as “unwanted”*	*Too many*	*63% (473)*	*21% (46)*	*81% (427)*
		*Poor decision*	*15% (116)*	*41% (91)*	*5% (25)*
		*Unspecified*	*4% (31)*	*12% (26)*	*1% (5)*
		*Other*	*17% (131)*	*26% (58)*	*14% (73)*
	**Changed circumstances**	**Total-Changed circumstances**	**20% (373)**	**29% (326)**	**6% (47)**
	*Proportion of total surrendered for “changed circumstances”*	*Moving/Travelling*	*61% (227)*	*60% (194)*	*70% (33)*
		*Household dynamic*	*23% (85)*	*24% (79)*	*13% (6)*
		*New Baby*	*5% (17)*	*5% (16)*	*2% (1)*
		*Unspecified*	*12% (44)*	*11% (37)*	*15% (7)*
	**Financial**		**16% (304)**	**14% (152)**	**20% (152)**
	**Accommodation**	**Total-Accommodation**	**5% (100)**	**7% (83)**	**2% (17)**
	*Proportion of total surrendered for “Accommodation” reasons*	*Landlord will not allow ^1^*	*60% (60)*	*59% (49)*	*65% (11)*
		*Yard too Small*	*15% (15)*	*14% (12)*	*18% (3)*
		*Property unsuitable*	*14% (14)*	*13% (11)*	*18% (3)*
		*Owner in care*	*8% (8)*	*10% (8)*	*0% (0)*
		*Acquired without consent of household/landlord ^2^*	*3% (3)*	*4% (3)*	*0% (0)*
	**Owner Health**	**Total-Owner Health**	**5% (86)**	**7% (77)**	**1% (9)**
	*Proportion of total surrendered due to “Owner Health”*	*Owner Ill*	*66% (57)*	*65% (50)*	*78% (7)*
		*Owner Deceased*	*20% (17)*	*22% (17)*	*0% (0)*
		*Owner Allergic*	*14% (12)*	*13% (10)*	*22% (2)*
	**Commercial ^3^**		**<1% (1)**	**<1% (1)**	**0% (0)**
**Dog**			**14% (257)**	**22% (245)**	**2% (12)**
	**Behavior**	**Total-Behavior**	**13% (250)**	**22% (239)**	**1% (11)**
	*Proportion of total surrendered due to “Behavior”*	*Escaping*	*20% (49)*	*20% (48)*	*9% (1)*
		*Aggression-Animal*	*17% (42)*	*16% (39)*	*27% (3)*
		*Aggressive-People*	*13% (32)*	*13% (30)*	*18% (2)*
		*Aggressive-Human or Animal*	*2% (4)*	*2% (4)*	*0% (0)*
		*Predatory behavior*	*4% (10)*	*4% (9)*	*9% (1)*
		*Boisterous*	*18% (45)*	*18% (44)*	*9% (1)*
		*Barking*	*6% (14)*	*5% (13)*	*9% (1)*
		*Destructive*	*4% (10)*	*4% (10)*	*0% (0)*
		*Fear*	*4% (9)*	*3% (7)*	*18% (2)*
		*Inappropriate toileting*	*1% (3)*	*1% (3)*	*0% (0)*
		*Other*	*13% (32)*	*13% (32)*	*0% (0)*
	**Dog size**		**<1% (6)**	**1% (6)**	**0% (0)**
	**Dog Health**		**<1% (1)**	**0% (0)**	**<1% (1)**
**Other**			**<1% (4)**	**<1% (4)**	**0% (0)**
**Total dogs surrendered**			**100% (1876)**	**59% (1109)**	**41% (767)**

^1^ Assumed to be relinquished prior to tenancy. ^2^ Assumed to be relinquished after start of tenancy. ^3^. Dog was described as “no good for racing”, see Supplementary Materials, Table S2.

**Table 7:** Summary of age group and outcome of 11,967 dogs processed through RSPCA Queensland shelters in 2014.

Outcome	Total	Adult	Puppy
Reclaimed	32% (3775)	39% (3473)	10% (302)
Adopted	43% (5183)	34% (2989)	71% (2194)
Euthanized	14% (1619)	17% (1471)	5% (148)
In Shelter	7% (804)	6% (517)	9% (287)
Transfer Out	4% (440)	4% (360)	3% (80)
Unassisted death	1% (89)	<1% (31)	2% (58)
In Foster	<1% (20)	<1% (15)	<1% (5)
Other	<1% (37)	<1% (27)	<1% (10)
Total dogs unclaimed	68% (8192)	61% (5410)	90% (2782)
Unclaimed dogs adopted	63% (5183)	55% (2989)	79% (2194)
Unclaimed dogs euthanized	20% (1619)	27% (1471)	5% (148)
Surrendered dogs adopted	72% (1580)	63% (780)	83% (800)
Surrendered dogs euthanized	14% (317)	22% (271)	5% (46)
**Total**	**11,967**	**8883**	**3084**

**Table 8:** Reasons for euthanasia for 1619 dogs euthanized in RSPCA-QLD shelters in 2014.

Euthanasia Reason Category	Euthanasia Reason Sub-Category	Total	Adult	Puppy
**Behavior**	**Total-Behavior**	**53% (855)**	**56% (821)**	**23% (34)**
*Proportion of total euthanized for behavioral reasons:*	*Aggression-Directed towards Animals*	*50% (428)*	*50% (413)*	*44% (15)*
	*Aggression-Target not defined*	*16% (133)*	*16% (128)*	*15% (5)*
	*Aggression-Directed towards humans*	*15% (128)*	*15% (126)*	*6% (2)*
	*Personality-Fearful*	*16% (134)*	*15% (125)*	*26% (9)*
	*Personality-Other*	*2% (15)*	*2% (13)*	*6% (2)*
	*Escaping*	*2% (17)*	*2% (16)*	*3% (1)*
**Health**	**Total-Health**	**23% (378)**	**19% (279)**	**67% (99)**
*Proportion of total euthanized for health reasons:*	*Musculoskeletal*	*18% (67)*	*20% (55)*	*12% (12)*
	*Parvovirus*	*17% (64)*	*7% (19)*	*45% (45)*
	*Cancer*	*12% (47)*	*17% (47)*	*0% (0)*
	*Neurological*	*5% (19)*	*5% (15)*	*4% (4)*
	*Heartworm*	*4% (15)*	*5% (15)*	*0% (0)*
	*Cardiac*	*2% (8)*	*2% (6)*	*2% (2)*
	*Ear*	*2% (6)*	*2% (6)*	*0% (0)*
	*Skin*	*1% (5)*	*2% (5)*	*0% (0)*
	*Tick Paralysis*	*1% (4)*	*1% (3)*	*1% (1)*
	*Ocular*	*1% (3)*	*1% (3)*	*0% (0)*
	*Dental*	*1% (2)*	*1% (2)*	*0% (0)*
	*Unspecified*	*37% (138)*	*37% (103)*	*35% (35)*
**Owner Requested**		**20% (323)**	**21% (315)**	**5% (8)**
**Age**	**Total-Age**	**3% (56)**	**3% (50)**	**4% (6)**
*Proportion of total euthanized for age reasons:*	*Too old*	*89% (50)*	*100% (50)*	*0% (0)*
	*Too young*	*11% (6)*	*0% (0)*	*100% (6)*
**Restricted Breed**		**<1% (6)**	**<1% (6)**	**0% (0)**
**Feral/wild dog ^1^**		**<1% (1)**	**0% (0)**	**1% (1)**
**Total dogs euthanized**		**100% (1619)**	**91% (1471)**	**9% (148)**

^1^ The dog in this category was a crossbred puppy, with dingo listed as its primary breed.

## Supplementary Materials

The following are available online at www.mdpi.com/2076-2615/7/9/67/s1, Table S1. Original source of admission organized into seven categories, Table S2. Original surrender reasons organized into human or dog related factors, categories and subcategories, Table S3. Original outcomes organized into eight categories, Table S4. Original euthanasia reasons organized into categories and subcategories, Table S1. List of suburbs for postcodes listed in Table 3 [52].
