# Supplementary material for: Characteristics and Outcomes of Dogs Admitted into Queensland RSPCA Shelters
Source: Animals (Basel). 2017 Sep 1;7(9):67. doi: 10.3390/ani7090067 (PMC5615298; doi:10.3390/ani7090067)
Supplement: Supplementary file 1 [file animals-07-00067-s001.pdf]

Table S1. Original source of admission organized into seven categories.

Source of Admission Categories	Original RSPCA Categories	Definition
Council	Council	Dogs accepted by the RSPCA from municipal pounds under a municipal pound contract
Stray	Stray	Lost/found dogs presented by a member of the public who is not the owner, or an agent of the owner
Owner Surrender	Ambulance- Owner Surrendered<br>Humane officer surrendered<br>Owner surrender	Dogs presented to the shelter (or agent of the shelter) by the owner or agent of the owner
Euthanasia Request	Euthanasia Request<br>Humane officer- Euthanasia Request	Dogs presented to the shelter with the owner's request to euthanize
Humane Officer	Ambulance<br>Humane officer<br>Humane officer seized	Dogs presented to the shelter by an agent of the shelter, other than municipal pounds (i.e., inspectors, humane officers and animal ambulance officers)
Offspring	Cruelty case offspring<br>Foster offspring<br>Shelter offspring	Dogs born under shelter or foster care
Other	Bequest in shelter<br>Emergency boarding<br>Returns<br>Transfer in	Bequest in shelter: Dogs willed to the RSPCA by a deceased estate<br>Emergency boarding: Dogs under temporary care of the shelter due to owner's circumstances<br>Returns: Dogs adopted but returned to the shelter within a defined period<br>Transfer in: Dogs transferred from other shelters or organizations

Table S2. Original surrender reasons organized into human or dog related factors, categories and subcategories.

Human/Dog Related Factor	Surrender Reason Category	Surrender Reason Sub-Category	Original RSPCA Surrender Reason
Dog	Behavior	Aggression-Animal	Aggression to Animals
			Behavior/Temperament of Existing Pet
		Aggressive-Human or Animal	Food Protective
			Resource Guarding/Over Protective
		Aggressive-People	Intolerant of children
			Snapping at Children
			Aggression to People
		Barking	Barking
		Destructive	Chewing
			Destructive
			Digging Yard
		Escape	Digging Out
			Jumping The Fence
			Keeps Getting Out
		Fear	Noise/Storm Phobic
			Separation Anxiety

Human/Dog Related Factor	Surrender Reason Category	Surrender Reason Sub-Category	Original RSPCA Surrender Reason
			Timid/Fearful/Not Coping in Household
		House soiling	Inappropriate toileting
		Other	This pet does not get on with existing pet
		Boisterous	Boisterous
			Too Boisterous
		Predatory behavior	Attacking Livestock
			Killing Wildlife
	Dog Health		Animal Medical Condition - Blind
	Dog size		Dog got too Big
Human	Accommodation	Owner in care	Owner Going into Care Facility - no pets allowed
		Property unsuitable	Cannot Contain on Property (Council Regulations)
		Acquired without consent of household/landlord	Acquired without consent of household/landlord
		Landlord will not allow	Landlord will not allow
		Yard too Small	Yard too Small
	Changed circumstances	Household dynamic	Relation Split
			Change of Household dynamic
		Moving/Travelling	Moving House
			Moving Overseas/Interstate
			Travelling
			going on holidays
			Owner Homeless
		New baby	New Baby
		Unspecified	Personal Reasons: Change in circumstance
	Commercial		No Good For Racing
	Financial		Can't Afford
			Cannot Afford Desexing (for registration)
			Cannot afford vet fees
			Surrender to Council-cannot afford fees
	Owner Health	Owner Allergic	Allergic
		Owner Ill	Owner-Ill Health
		Owner Deceased	Owner Deceased
	Unwanted	Other	Previous owner left over four weeks ago
			Unwanted
			Formerly Stray
			Unwanted Gift
		Poor decision	Impulse Buy
			No Time
			Poor choice
		Too many	Last of Litter
			Too Many Dogs
			Unwanted litter
		Unspecified	Owner surrendered to external stakeholder
			Surrendered to Council-reason not specified
Other	Other		Other

Table S3. Original outcomes organized into eight categories [52].

Outcome Categories	Original RSPCA Categories
Adopted	Adopted

	Adopted offsite<br>Adopted to finder
Reclaimed	Emergency boarding returned to owner<br>Reclaimed<br>Redemption (offsite)<br>Released to owner at vet<br>Returned home<br>Returned protective custody animal<br>Returned surrender
Euthanized	Euthanasia in field<br>Euthanized<br>Euthanized by offsite vet<br>Euthanasia/cremation request
In Shelter	In shelter<br>Available for adoption<br>Available for adoption- no web presence<br>Court hold<br>Police hold<br>Sanctuary/life resident<br>Stray hold<br>Under vet care<br>Unresolved file
In Foster	Available for adoption- In foster<br>Awaiting spay/neuter- In foster<br>In foster
Unassisted death	Deceased<br>Unassisted death<br>Unassisted death-in foster
Transferred out	Transfer out
Other	Escaped<br>Matched<br>Possible match<br>Privately rehomed-new owner unknown<br>Stolen

Table S4. Original euthanasia reasons organized into categories and subcategories.

Euthanasia Reason Category	Euthanasia Reason Sub-Category	Original RSPCA Euthanasia Reason
Age	Too old	Old
	Too young	Too young < six weeks or under 500grams
Behavior	Aggression-Directed towards animals	Aggression-Inter Cat
		Aggressive-Dog
	Aggression-Target not defined	Aggression-Fence Aggressive
		Aggression-Redirected Aggression
		Declared Dangerous
		Guarding Behaviors-
		Resource Guarding
		Untrustworthy
		Dominance Aggression
	Aggression-Directed towards humans	Aggressive-Humans
		Fearful/aggressive
	Escaping	Escape Behavior
	Personality-Other	Compulsive; obsessive; stereotypic behavior
	Personality-Fearful	Hyper Reactivity To Stimuli
		Separation Anxiety
		Timid/fearful With Accompanying Anxiety

Euthanasia Reason Category	Euthanasia Reason Sub-Category	Original RSPCA Euthanasia Reason
		Under socialized-Not Coping
		Not Coping in Pens-Pen Running
Feral/Wild dog	Feral/Wild dog	Feral
Health	Cancer	Malignancy (cancer)
	Cardiac	Cardiac Disease
	Heartworm	Heartworm Positive
	Dental	Dental Disease
	Ear	Chronic Ear Infection/Problems
	Musculoskeletal	Injured
		Orthopedic Problems
	Neurological	Neurological Problems
		Paralysis
		Neurological
	Ocular	Blind
	Parvovirus	Parvo
		Parvo Contact
	Skin	Excessive Scarring
		Hair loss-Demodex
		Hair loss-Nonspecific
		Hair loss-Ringworm
	Tick	Tick Paralysis
	Unspecified	Humane Grounds
		Sick
Owner requested	Owner Requested	Owner Requested (Please specify in text box)
Restricted Breed	Restricted Breed	Restricted Breed

Table S5. List of suburbs for postcodes listed in Table 3 **[52].**

Postcode	Suburbs
4019	Margate Beach, Woody Point, Clontarf Beach, Clontarf, Margate, Clontarf Dc
4021	Kippa-Ring
4076	Darra, Wacol
4300	Springfield, Springfield Central, Gailes, Goodna, Springfield Lakes, Augustine Heights, Brookwater, Bellbird Park, Carole Park, Camira
4350	Finnie, East Toowoomba, Gowrie Mountain, Glenvale, Darling Heights, Cranley, Drayton North, Drayton, Charlton, Centenary Heights, Cotswold Hills, Clifford Gardens, Blue Mountain Heights, Athol, Middle Ridge, Kearneys Spring, Harristown, Harlaxton, Newtown, Mount Rascal, Mount Lofty, Mount Kynoch, Prince Henry Heights, Northpoint, Northlands, North Toowoomba, South Toowoomba, Rockville, Redwood, Rangeville, Toowoomba City, Toowoomba Dc, Southtown, Toowoomba, Toowoomba Village Fair, Toowoomba West, Toowoomba East, Toowoomba South, Wellcamp, Westbrook, Top Camp, Torrington, Wyalla Plaza, Wilsonton, Wilsonton Heights
4500	Cashmere, Clear Mountain, Joyner, Strathpine, Bray Park, Brendale, Brendale Bc, Brendale Dc, Warner, Strathpine Centre
4501	Lawnton
4502	Petrie
4503	Kurwongbah, Kallangur, Griffin, Dakabin, Whiteside, Murrumba Downs
4504	Narangba
4505	Burpengary, Burpengary East, Burpengary Dc
4506	Morayfield, Moorina
4508	Deception Bay

4510	Meldale, Donnybrook, Rocksberg, Moodlu, Upper Caboolture, Toorbul, Beachmere, Caboolture BC, Caboolture South, Bellmere, Caboolture
4562	Weyba Downs, Verrierdale, Doonan, Belli Park, Eumundi, Eerwah Vale
4570	Gilldora, Glanmire, East Deep Creek, Fishermans Pocket, Dagun, Downsfield, Corella, Curra, Gympie, Gympie Dc, Greens Creek, Gunalda, Glenwood, Goomboorian, Glastonbury, Glen Echo, Bollier, Bells Bridge, Bella Creek, Beenaam Valley, Banks Pocket, Araluen, Anderleigh, Amamoor Creek, Coondoo, Coles Creek, Chatsworth, Cedar Pocket, Canina, Calico Creek, Calgoa, Brooloo, Amamoor, Wolvi, Woolooga, Widgee Crossing South, Wilsons Pocket, Woondum, The Palms, The Dawn, Tandur, Tamaree, Tuchekoi, Traveston, Toolara Forest, Theebine, Veteran, Upper Kandanga, Upper Glastonbury, Two Mile, Widgee Crossing North, Widgee, Wallu, Victory Heights, Miva, Monkland, Mooloo, Mothar Mountain, Munna Creek, Nahrunda, Neerdie, Neusa Vale, North Deep Creek, Paterson, Pie Creek, Ross Creek, Scotchy Pocket, Scrubby Creek, Sexton, Southside, Jones Hill, Imbil, Kandanga Creek, Kandanga, Kia Ora, Kanigan, Lagoon Pocket, Kybong, Langshaw, Lake Borumba, Lower Wonga, Long Flat, Marys Creek, Marodian, Melawondi, Mcintosh Creek
4580	Tin Can Bay, Cooloola, Cooloola Cove
4610	Kingaroy, Ironpot, Inverlaw, Hodgleigh, Haly Creek, Gordonbrook, Goodger, Ellesmere, Wattle Grove, Taabinga, Memerambi, Mannuem, Kumbia, Kingaroy Dc, Ballogie, Benair, Alice Creek, Dangore, Durong, Corndale, Crawford, Chahpingah, Coolabunia, Booie, Boyneside
4615	Wengenville, Wattle Camp, Tarong, South Nanango, Wyalla, East Nanango, Bullcamp, Glan Devon, Elgin Vale, Brooklands, Barker Creek Flat, Runnymede, Pimpimbudgee, South East Nanango, Sandy Ridges, Kunioon, Johnstown, Nanango, Maidenwell
4670	Windermere, Welcome Creek, Woongarra, Winfield, Sharon, Rubyanna, South Kolan, South Bingera, Thabeban, Svensson Heights, Watalgan, Walkervale, Abbotsford, Bargara, Avondale, Bucca, Branyan, Ashfield, Alloway, Avoca, Avenell Heights, Bundaberg West, Burnett Heads, Bundaberg North, Bundaberg South, Bundaberg Dc, Bundaberg East, Bundaberg, Bundaberg Central, Fairymead, Givelda, Elliott, Elliott Heads, Coral Cove, Electra, Calavos, Coonarr, Millbank, Meadowvale, Kinkuna, Kepnock, Kensington, Kalkie, Innes Park, Gooburrum, Qunaba, Pine Creek, Oakwood, Norville, Mullett Creek, Moorland, Moore Park Beach, Mon Repos
4680	Boyne Valley, Boyne Island, Builyan, Boynedale, Barney Point, Benaraby, Beecher, Glen Eden, Heron Island, Iveragh, Kin Kora, Gladstone Bc, Gladstone Central, Gladstone Dc, Gladstone Harbour, Clinton, Curtis Island, Diglum, Gladstone, Burua, Byellee, Callemondah, Calliope, West Gladstone, Ubobo, Toolooa, Telina, Taragoola, Tannum Sands, Tablelands, Sun Valley, South Trees, South Gladstone, South End, River Ranch, O'connell, New Auckland, Mount Alma, Kirkwood, Wurdong Heights, West Stowe, Wooderson
4810	Belgian Gardens, Townsville City, Townsville DC, Town Common, Townsville, Townsville MC, West End, North Ward, Pallarenda, Cape Cleveland, Castle Hill, Shelly Beach, South Townsville, Railway Estate, Rowes Bay
4812	Hyde Park Castletown, Hyde Park, Hermit Park, Gulliver, Rosslea, Pimlico, Mysterton, Mundingburra, Currajong
4814	Mount Louisa, Murray, Thuringowa Dc, Vincent, Aitkenvale, Annandale, Cranbrook, Douglas, Garbutt, Garbutt East, Heatley
4815	Rasmussen, Pinnacles, Condon, Granite Vale, Gumlow, Kelso
4817	Thuringowa Central, Rangewood, Kirwan, Hervey Range, Bohle Plains, Alice River
4818	Black River, Beach Holm, Saunders Beach, Shaw, Toolakea, Yabulu, Jensen, Lynam, Mount Low, Mount St John, Burdell, Bushland Beach, Cosgrove, Deeragun, Blue Hills, Bluewater, Bluewater Park, Bohle
